# MiR408-*SmLAC3* Module Participates in Salvianolic Acid B Synthesis in *Salvia miltiorrhiza*

**DOI:** 10.3390/ijms22147541

**Published:** 2021-07-14

**Authors:** Haolan Zou, Xiaorong Guo, Rao Yang, Shengsong Wang, Lin Li, Junfeng Niu, Donghao Wang, Xiaoyan Cao

**Affiliations:** Key Laboratory of the Ministry of Education for Medicinal Resources and Natural Pharmaceutical Chemistry, National Engineering Laboratory for Resource Development of Endangered Crude Drugs in Northwest of China, Shaanxi Normal University, Xi’an 710062, China; zhl2019@snnu.edu.cn (H.Z.); guoxiaorong@snnu.edu.cn (X.G.); yangrao@snnu.edu.cn (R.Y.); shengsongwang@snnu.edu.cn (S.W.); shidalilin@snnu.edu.cn (L.L.); niujunfeng@snnu.edu.cn (J.N.); wangdonghao@snnu.edu.cn (D.W.)

**Keywords:** *Sm*-miR408, *SmLAC3*, salvianolic acid B, *Salvia miltiorrhiza*

## Abstract

MicroRNAs (miRNAs) are important regulators of gene expression involved in plant development and abiotic stress responses. Recently, miRNAs have also been reported to be engaged in the regulation of secondary plant metabolism. However, there are few functional studies of miRNAs in medicinal plants. For this study, we obtained *Sm*-miR408 interference lines to investigate the function of *Sm*-miR408 in a medicinal model plant (*Salvia miltiorrhiza*). It was found that inhibiting the expression of *Sm*-miR408 could increase the content of salvianolic acid B and rosmarinic acid in the roots. The *SmLAC3* and *Sm*-miR408 expression patterns were analyzed by qRT-PCR. A 5’ RLM-RACE assay confirmed that *Sm*-miR408 targets and negatively regulates *SmLAC3*. Moreover, the overexpression of *SmLAC3* in *S. miltiorrhiza* promoted the accumulation of salvianolic acids in the roots. Furthermore, the lignin content of the roots in overexpressed *SmLAC3* lines was decreased. Taken together, these findings indicated that *Sm*-miR408 modulates the accumulation of phenolic acids in *S. miltiorrhiza* by targeting *SmLAC3* expression levels.

## 1. Introduction

MicroRNAs (miRNAs) comprise a type of non-coding small RNA (typically 20–24 nucleotides in length) [1] that regulate the expression of target genes at the post-transcriptional level in animals and plants [2]. MiRNA can directly cleave target gene sequences when it shows a high degree of sequence complementarity to its target mRNA [3,4]. In the case of incomplete complementation, miRNA specifically binds to the 3’ untranslated region of the target mRNA to inhibit translation [5]. With the further study of plant miRNAs, researchers have found that this class of regulatory molecule exhibits spatiotemporal and tissue specificity while playing essential roles in plant growth and development and responds to environment stimuli [6]. Plant miRNAs have been demonstrated to be involved in the regulation of seed development [7], leaf morphogenesis [8,9] as well as floral and root development [10,11,12]. Their transcription can also be regulated to further alter the expression of their target genes in response to drought [13], oxidative stress [14], copper deficiency [15], and other stressors [16]. 

The targets of plant miRNAs also contain genes that encode transcription factors to regulate the accumulation of secondary metabolites, which suggests that miRNAs participate in the regulation of secondary plant metabolism. In *Arabidopsis*, the miR163 target genes are members of the methyltransferase gene family, which is involved in the biosynthesis of plant secondary metabolites. They affect the conversion of farnesoic acid to methyl farnesoate, which perturbs insect growth and development by negatively regulating the expression of farnesoic acid methyltransferase [17]. MiR156-targeted SPLs can negatively regulate anthocyanin biosynthesis through the disruption of the MYB-bHLH-WD40 transcriptional activation complex, which activates anthocyanin biosynthetic genes [18]. The overexpression of miR8154 and miR5298b in the *Taxus* cell line results in the upregulation of some key taxol genes, phenylpropane, and flavonoid biosynthesis such as taxadiene synthase, phenylalanine ammonia lyase, and chalcone synthase, which indicates that these miRNAs have some influence on secondary metabolism [19].

The miR408 family targets genes that encode several small blue proteins and laccase (a group of blue oxidases), both of which are blue copper proteins [20,21]. MiR408 maintains the stability of the internal environment by regulating the concentration of copper ions that participate in processes such as photosynthesis and cell wall lignification. Previous studies have shown that the expression of miR408 is not only affected by copper ion concentrations, but also by abiotic stress, which suggests that miR408 plays a certain role in the responses of plants to abiotic stresses [22,23,24]. Moreover, miR408 is involved in the regulation of secondary metabolism. The anthocyanin content of the transgenic seedlings overexpressing miR408 was 7–8 times that of wild type *Arabidopsis* [25]; however, the regulatory mechanism is unclear. Whether miR408 participates in the regulation of secondary metabolism in other plants, particularly in medicinal plants, is unknown. Although we investigated the functions of miR408 from *Salvia miltiorrhiza* (a model medicinal plant) by overexpressing *Sm-MIR408* in *Nicotiana benthamiana* and found that it positively influences responses to salt tolerance [26], the functions of miR408 in *S. miltiorrhiza* have not been reported as yet.

Laccase (EC 1.10.3.2) is a type of blue multicopper oxidase that contains four copper atoms and has been found to be widespread in plants, bacteria, fungi, and insects [27]. Research over the years has revealed that laccase in plants is associated with lignin polymerization [28] and flavonoid biosynthesis [29]. For instance, a knockout of *AtLAC11* along with either *AtLAC4* or *AtLAC17* and a *lac11lac4lac17* triple mutant significantly reduced the lignin content [30]. *AtLAC15* mutants accumulated more epicatechin monomers and soluble proanthocyanidins than wild-type seeds, suggesting that *LAC15* may contribute to the oxidative polymerization of flavonoids [31]. In exploring the function of cotton LACs, researchers found that GaLAC1 promotes the conversion of sinapic acid to a monolactone-type dimer [32]. Nevertheless, the biochemical or physiological functions of laccases in plants remain largely unclear, and related reports are rare.

**Figure 1:**
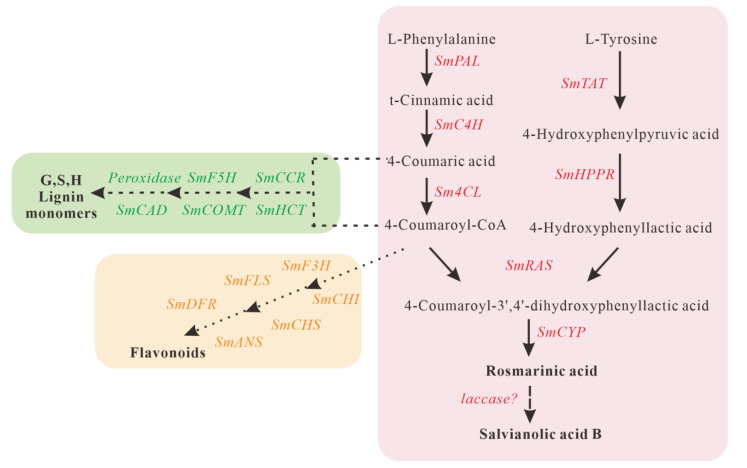
The proposed biosynthetic pathways for phenolic acids, flavonoids, and lignin in *Salvia miltiorrhiza*. *PAL*, phenylalanine ammonia lyase; *C4H*, cinnamate 4-hydroxylase; *4CL*, hydroxycinnamate-CoA ligase; *TAT*, tyrosine aminotransferase; *HPPR*, hydroxyl phenylpyruvate reductase; *RAS*, rosmarinic acid synthase; *CYP*, cytochrome P450 enzymes; *CHI*, chalcone isomerase; *F3H*, flavanone 3-hydroxylase; *CHS*, chalcone synthase; *FLS*, flavonol synthase; *DFR*, dihydroflavonol 4-reductase; *ANS*, anthocyanin synthase; *HCT*, hydroxyl cinnamoyl transferase; *CCR*, cinnamoyl-CoA reductase; *COMT*, caffeic acid O-methyltransferase; *F5H*, ferulate 5-hydroxylase; *CAD*, cinnamyl alcohol dehydrogenase. Solid lines indicate the step is specific. Dashed lines indicate the step is uncertain or multiple enzymatic steps are represented.

Salvianolic acid B (SalB) is one of the main active ingredients of *S. miltiorrhiza* and is considered a dimer of rosmarinic acid (RA). The biosynthetic pathway of RA in plants has been demonstrated (Figure 1); however, how RA is converted into SalB is still unknown. It has been speculated that laccases in *S. miltiorrhiza* might catalyze RA to SalB [33,34]. In the present study, the function of *Sm*-miR408 was investigated by knocking down its expression in *S. miltiorrhiza*. *SmLAC3* was verified as the authentic target of *Sm*-miR408 using 5′RLM-RACE. The enhanced expression of *SmLAC3* was achieved through gain-of-function analysis. The contents of SalB and RA were increased in both miR408-suppressed and *SmLAC3*-overexpressed transgenic lines. The results suggested that *Sm*-miR408 regulated the biosynthesis of SalB using its target gene *SmLAC3*. To the best of our knowledge, this is the first functional study of miRNA in *S. miltiorrhiza* and the first report of miRNA being involved in the regulation of the active ingredients in a medicinal plant.

## 2. Results

### 2.1. MiR408 Negatively Regulates the Synthesis of SalB and RA in S. miltiorrhiza

**Figure 2:**
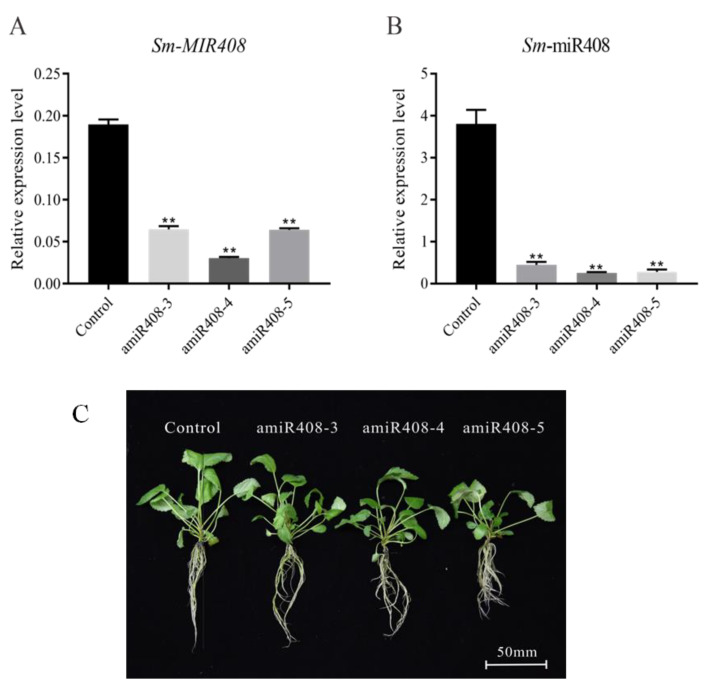
Identification and phenotype of the miR408-suppressed transgenic *Salvia miltiorrhiza*. (**A**) Expression level of the precursor miR408. (**B**) Expression level of mature miR408. (**C**) Phenotype of the miR408-suppressed transgenic lines (amiR408-3, amiR408-4, and amiR408-5) and the control. All data are means of three independent experiments, with error bars representing SD. Significant differences in comparison with the control were determined by Dunnett’s multiple comparison test (** *p* < 0.01).

To investigate the function of miR408 in *S. miltiorrhiza*, we obtained miR408-suppressed transgenic plantlets (amiR408) via *Agrobacterium*-mediated transformation. After selective culturing on an herbicide medium, three transgenic lines (amiR408-3, amiR408-4, and amiR408-5) were used to detect the expression of miR408. Quantitative reverse transcription PCR (qRT-PCR) results indicated that the expression levels of both the precursor and mature miR408 were significantly suppressed in the transgenic plants compared to that of the control (Figure 2). In comparison to the control, the *Sm*-miR408 interference lines did not show an obvious phenotype change (Figure 2). Two transgenic lines, amiR408-3 and amiR408-4, were used for the following experiments.

**Figure 3:**
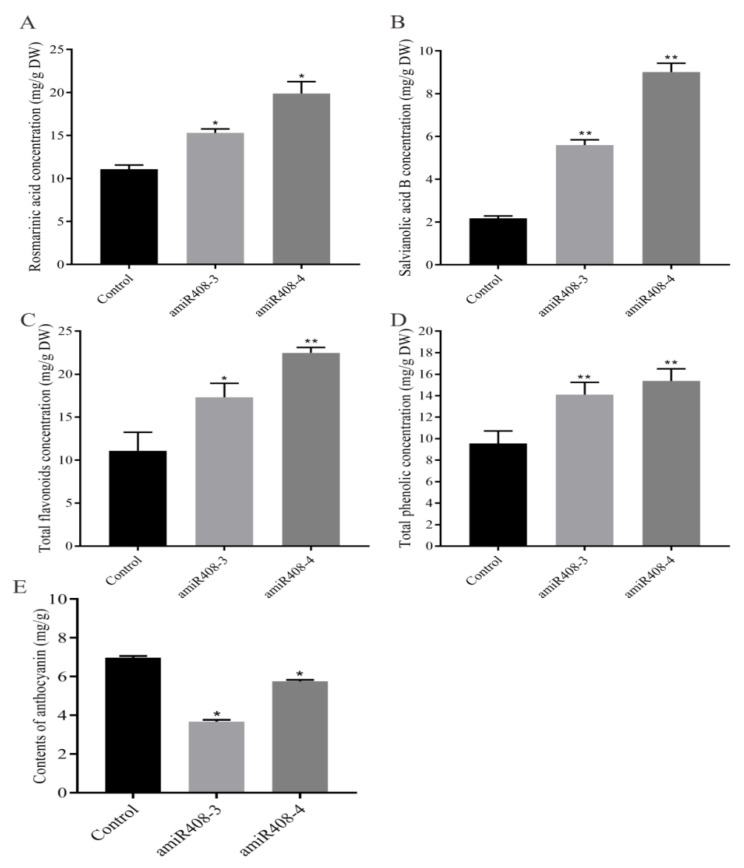
Detection of rosmarinic acid (**A**), salvianolic acid B (**B**), total flavonoids (**C**), total phenolic (**D**), and anthocyanin (**E**) content in transgenic (amiR408) and control lines. All data represent mean ± SD of three biological replicates. Significant differences in comparison with the control were assessed with Dunnett’s multiple comparison test (** *p* < 0.01; * *p* < 0.05).

To determine whether knocking down the expression of miR408 would affect the synthesis of phenolic acids in *S. miltiorrhiza*, we tested the concentrations of RA and SalB via high performance liquid chromatography (HPLC). The results showed that both RA and SalB were significantly increased compared to those of the untransformed control. As shown in Figure 3A, the quantity of RA was 1.4 and 1.82 times higher in amiR408-3 and amiR408-4, respectively, while the content of SalB was approximately 2.52 times and 4.11 times higher than those of the control (Figure 3B).

We also undertook objective of detecting the total phenolics and total flavonoids, both of which share an upstream core phenylpropanoid metabolism with SalB. The results revealed that the levels of total phenolics and total flavonoids were significantly higher in the transgenic lines than in the control (Figure 3C,D). Compared to the control, the content of total phenolics increased by 1.42 times and 1.61 times in amiR408-3 and amiR408-4, respectively, and the total flavonoids increased by 1.59 times and 2.08 times, respectively. In addition, we measured the anthocyanin content in these lines and found that its level in the transgenic lines was lower than in the control (Figure 3E).

**Figure 4:**
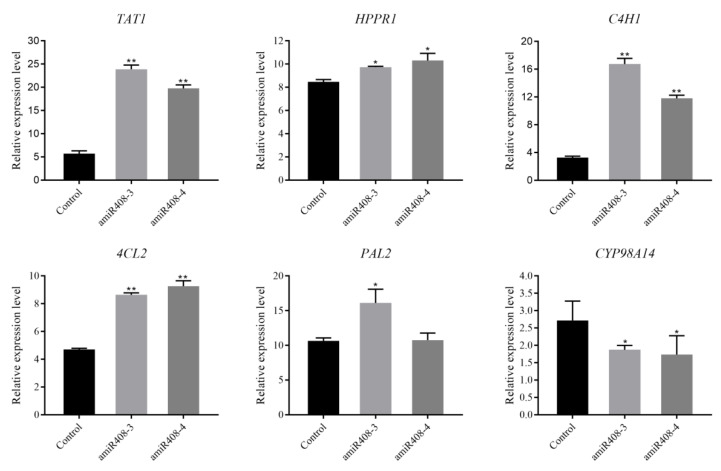
Relative expression levels of the key enzyme genes involved in phenolic acid biosynthetic pathway in the transgenic (amiR408) and control lines. All data are means of three independent experiments, with error bars representing SD. Significant differences in comparison with the control were determined by Dunnett’s multiple comparison test (** *p* < 0.01; * *p* < 0.05).

The above results demonstrated that inhibiting the expression of miR408 leads to higher phenolic acid levels. Furthermore, we examined the transcript levels of six genes in the RA synthetic pathway and the results indicated that most of those genes, including *SmTAT1, SmPAL2, SmC4H1, Sm4CL2,* and *SmHPPR1,* were significantly induced in the amiR408 lines (Figure 4). In particular, the transcript level of *TAT1* was increased by 4.2- and 3.5 times in amiR408-3 and amiR408-4, respectively. All of these findings demonstrated that knocking down miR408 induces the expression of enzyme genes in the phenolic acid biosynthetic pathway, leading to the enhancement of RA and SalB content.

### 2.2. SmLAC3 Is the Target of Sm-miR408

It is well known that miRNAs exert their functions by inhibiting the expression of target genes, and miRNAs need to be strictly complementary with their target genes in order to cleave at the pairing sites [35,36]. To reveal the target of *Sm*-miR408, we predicted its potential targets through the website psRNATarget (http://plantgrn.noble.org/psRNATarget/, accessed in 12 May 2018) [37]. Under relatively strict parameters, *SmLAC3*, *SmCYP72A327*, and *SmBRI-like3*, which encode laccase, the cytochrome P450 enzyme, and the putative receptor-like protein kinase brassinosteroid insensitive 3, respectively, were predicted as the putative targets of *Sm*-miR408.

**Figure 5:**
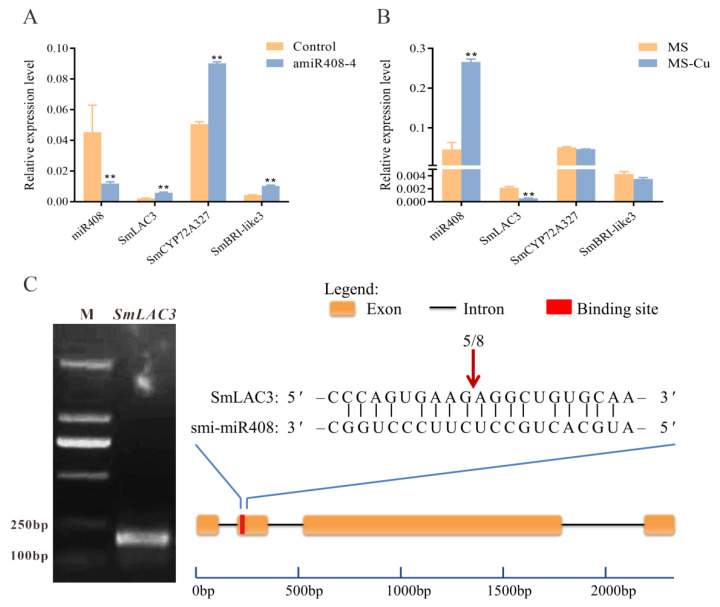
Validation of *Sm*-miR408 target genes. (**A**) Relative expression levels of the Sm-miR408 putative target genes in amiR408 lines compared to control lines. (**B**) Relative expression levels of the *Sm*-miR408 putative target genes under normal and copper starvation conditions. (**C**) *Sm*-miR408 cleavage site in *SmLAC3* mRNA validated by 5’ RLM-RACE. For RT-qPCR, all data represent the means of three biological replicates, with error bars indicating SD. Significant differences were determined by Student’s *t*-test (** *p* < 0.01).

To validate the targets of *Sm*-miR408, we analyzed the transcription levels of *Sm*-miR408 and its putative target genes using qRT-PCR. First, their transcripts were detected in the control and amiR408 lines. As shown in Figure 5A, compared to the control, the expression levels of the three putative targets were significantly up-regulated in the amiR408 lines. The abundance of miR408 is negatively regulated by the availability of copper [15] and has been annotated in more than 20 plant species [38]. Consequently, we detected the expression levels of *Sm*-miR408 and its putative targets in *S. miltiorrhiza* under normal and copper starvation conditions (Figure 5B). The results indicated that *Sm*-miR408 accumulated to a higher level under copper-deficient conditions. Among the three putative target genes, compared to the normal condition, only *SmLAC3* was significantly down-regulated under copper starvation conditions. Hence, qRT-PCR analysis revealed that *SmLAC3* was the most prospective target of *Sm*-miR408.

The 5’ RNA ligase-mediated rapid amplification of cDNA ends (5’ RLM-RACE) is a PCR-based technique that has been widely employed to validate the cleavage sites of target genes using miRNA [39,40]. Here, we performed 5’RLM-RACE to detect whether *Sm*-miR408 cleaved its target genes. The results indicated that of the eight randomly selected clones, five possessed the 5′ ends of the cleaved fragments at the same site as the *SmLAC3*. The cleavage site for *SmLAC3* resided between the 11th and 12th nucleotides of smi-miR408 (Figure 5C); however, *SmCYP72A327* and *SmBRI-like3* were not cleaved by miR408. These results indicated that *SmLAC3* was an authentic target gene of miR408, and its expression level was contingent on the miR408-directed cleavage modes of the miR408.

### 2.3. Expression Profiles of SmLAC3 in S. miltiorrhiza

**Figure 6:**
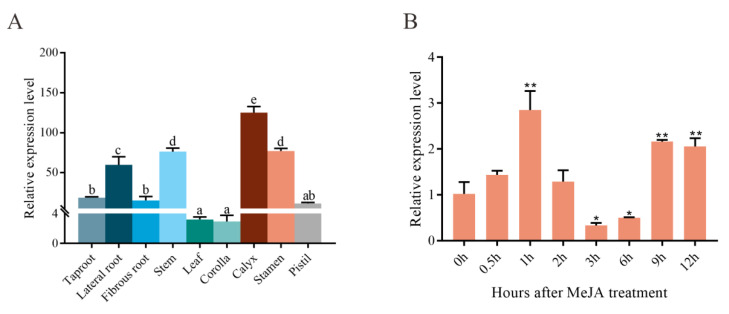
Expression patterns of *SmLAC3.* (**A**) qRT-PCR analysis of *SmLAC3* expression in various *S. miltiorrhiza* tissues. Different lowercase letters indicate significant differences at *p* < 0.05 by one-way ANOVA with Tukey’s multiple comparisons test. (**B**) *SmLAC3* expression in response to a 100 μM MeJA treatment. All data represent the means of three biological replicates, with error bars indicating SD. Significant differences were determined by Dunnett’s multiple comparison test (** *p* < 0.01; * *p* < 0.05).

We investigated the spatial expression patterns of *SmLAC3* in various tissues using qRT-PCR analysis. As shown in Figure 6A, *SmLAC3* was expressed in all of the tested tissues, and it exhibited the highest level in the calyxes, followed by the stems and stamen, with the lowest transcript level in the corollas and leaves.

Most genes in phenolic acid biosynthesis can be induced by methyl jasmonate (MeJA), which leads to the accumulation of SalB [41,42]. We detected the expression profile of *SmLAC3* under the condition of MeJA treatment. The results indicated that *SmLAC3* responded to the MeJA treatment (Figure 6B), and significant expression was induced at 1 h, 9 h, and 12 h following the MeJA treatment, but was significantly suppressed at 3 h and 6 h. These fluctuating changes showed that the expression profile of *SmLAC3* was complicated when treated by MeJA.

### 2.4. Overexpression of SmLAC3 Promotes the Biosynthesis of SalB and RA in S. miltiorrhiza

**Figure 7:**
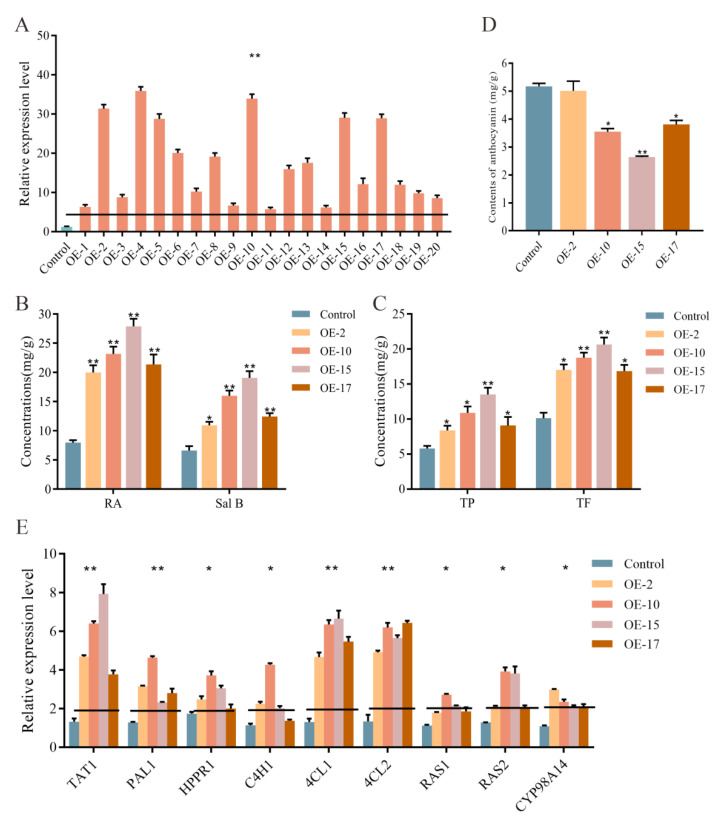
Effects of overexpressed *SmLAC3* on the phenolic acid biosynthesis pathway. (**A**) qRT-PCR analysis of *SmLAC3* expression in transgenic and control lines. (**B**) Concentrations of RA and SalB in the roots of *SmLAC3*-OE and control lines. (**C**) Concentrations of TP and TF in the roots of *SmLAC3*-OE and control lines. (**D**) Detection of anthocyanin content in *SmLAC3*-OE and control lines. (**E**) Relative expression levels of genes involved in the *SmLAC3*-OE phenolic acid pathway and control lines. All data are the means of three independent experiments, with error bars representing SD. Significant differences in comparison with the control were determined by Dunnett’s multiple comparison test (** *p* < 0.01; * *p* < 0.05).

To elucidate whether *SmLAC3* affects the synthesis of SalB and RA, we generated transgenic plants that overexpressed *SmLAC3* (*SmLAC3*-OE). We detected the *SmLAC3* expression level in these OE lines using qRT-PCR, with the results showing that the transcripts of *SmLAC3* in all of the transgenic lines were significantly higher than those in the control (Figure 7A). Four *SmLAC3*-OE lines with higher expression levels (OE-2, OE-10, OE-15, and OE-17) were selected for subsequent experiments. As anticipated, a significant increase in the concentrations of both RA and SalB was observed in the roots of the *SmLAC3*-OE lines.

The quantities of RA were 2.44 times, 2.93 times, 3.48times, and 2.62times higher in OE-2, OE-10, OE-15, and OE-17, respectively, than that of the control, while the amount of SalB was approximately 1.82 times, 2.79times, 3.42times, and 2.13times higher, respectively, than that of the control (Figure 7B). Moreover, the total phenolic and total flavonoid concentrations of the *SmLAC3*-OE lines were significantly increased when compared to the control. Between the four lines, OE-15 showed the highest degree of change with the levels of total phenolics and total flavonoids being 2.50 times and 2.06 times higher, respectively (Figure 7C). We also detected the content of anthocyanin in two SmLAC3-OE lines, and the results showed that its level was lower than that in the control lines (Figure 7D).

We further examined the transcript levels of nine enzyme genes of the biosynthetic pathway for phenolic acids in the *SmLAC3*-OE lines using qRT-PCR, with the results shown in Figure 7E. These genes were all significantly up-regulated in the *SmLAC3*-OE lines. Among the tested genes, the expression levels of *SmTAT1*, *Sm4CL1*, and *Sm4CL2* exhibited more pronounced changes. In OE-2, OE-10, OE-15, and OE-17, the *SmTAT1* expression levels were up-regulated by 4.53 times, 6.35 times, 7.92 times, and 3.31 times, respectively, when compared to the empty vector control lines; *Sm4CL1* was up-regulated by 4.81 times, 6.13 times, 6.38 times, and 5.42 times, respectively; and *Sm4CL2* was upregulated by 5.09 times, 6.21 times, 5.36 times, and 6.32 times, respectively. These data suggest that the overexpression of *SmLAC3* in transgenic *S. miltiorrhiza* promoted the transcription of genes for the biosynthesis of phenolic acids and increased the concentrations of RA and SalB.

### 2.5. Overexpressing SmLAC3 Decreases the Lignin Content in the Roots

Previous studies have shown that the laccase of plants can promote the synthesis of lignin [43,44]. Lignin and phenolic acids share a common upstream phenylpropanoid pathway [34,45]. We were curious as to the changes in the lignin content in the roots of *SmLAC3*-OE lines; thus, the safranin staining method was used to explore whether the hyper-accumulation of *SmLAC3* alters the lignification of cell walls in the roots of *S. miltiorrhiza*.

**Figure 8:**
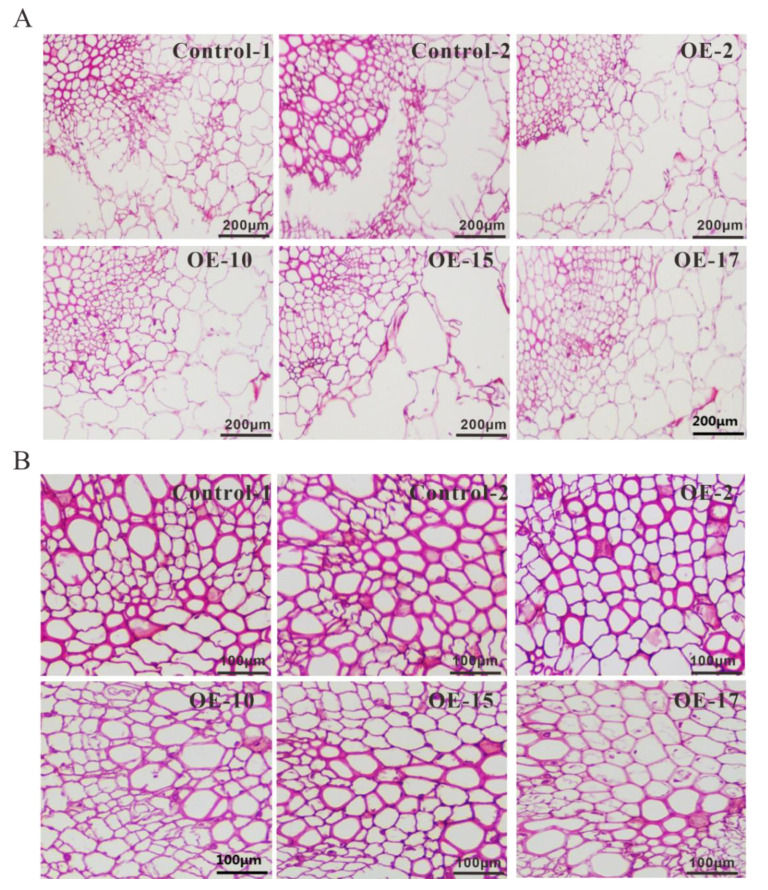
Histochemical stain of lignin in the roots of *SmLAC3*-OE and control lines. (**A**) Lignin staining in the roots of the control and transgenic lines. Scale bar denotes 200 μm. (**B**) Magnified views of images in A. Scale bar denotes 100 μm.

As shown in Figure 8, compared to the control, the staining of the *SmLAC3*-OE lines was lighter and less vivid, which indicated that less lignin was deposited in the roots of the *SmLAC3*-OE lines. We further determined the lignin content in the *SmLAC3*-OE lines and the control, and the result showed that the lignin content in the roots of the *SmLAC3*-OE lines was lower than that of the control (Table 1), which was consistent with the staining results. We speculated that the decrease of lignin in the *SmLAC3*-OE lines might be caused by a reduction of metabolic flux into the lignin biosynthesis branch due to the significant increase of phenolic acid biosynthesis.

## 3. Discussion

### 3.1. MiR408 Has Multiple Functions in Plants

MiR408 is a class of highly conserved miRNA in plants that is composed of 21 nucleotides [46]. Since its initial discovery in *Arabidopsis thaliana*, miR408 has been found in a variety of plants. Current research on miR408 in plants has focused on its important roles in plant growth and development as well as in responses to abiotic stress. In *A. thaliana*, the upregulation of miR408 enhanced plant tolerance to cold, salinity, and oxidative stress and delayed plant senescence [21]. The overexpression of miR408 in rice led to a higher pollen germination rate [47]. Overexpressed miR408 also translated to an improvement in photosynthetic performance, a higher rate of vegetative growth, and increased seed yields in *A. thaliana*, *Oryza sativa*, and *Nicotiana tabacum* [23]; however, the overexpression of miR408 in *Ipomoea batatas* resulted in a semi-dwarf phenotype [48]. In the present study, miR408-suppressed *S. miltiorrhiza* showed no obvious phenotype changes.

Furthermore, miR408 was reported to be involved in the regulation of secondary metabolism, where overexpressed miR408 in *Arabidopsis* significantly promoted the accumulation of anthocyanin [25]. Here, we measured the levels of total phenolics, total flavonoids, and anthocyanin in the miR408-suppressed transgenic *S. miltiorrhiza* (Figure 3). It was found that the concentrations of total flavonoids and total phenolics increased, while the anthocyanin content in the miR408 interference lines decreased in contrast to the control, which was consistent with the results in *A. thaliana* (Figure 3C).

As a model medicinal plant, *S. miltiorrhiza* contains several representative active ingredients, such as SalB and RA. We determined their contents in miR408 interference lines and found that both SalB and RA were significantly increased (Figure 3A). These results suggested miR408 affected not only the accumulation of anthocyanin in *S. miltiorrhiza*, but also the synthesis of salvianolic acids. To the best of our knowledge, this is the first report to articulate the functions of miRNA in *S. miltiorrhiza*.

### 3.2. Function of Laccase in S. miltiorrhiza

Laccase is extensively distributed across various organisms in nature, encompassing fungi, bacteria, plants, and insects. Although the structures of laccases are similar in different species, their functions are not identical [49,50,51]. To date, research on laccase has mainly focused on fungal laccase, with few studies on the functions of laccase in plants. Most of laccases in plants are involved in lignification. Regulation of lignin synthesis plays an important role in maintaining the structural integrity of cell wall and improving plant defense [52]. Moreover, in some bioenergy crops, the change of lignin composition will affect the biosaccharification rate, affecting the economic value of the crops [52]. In *Gossypium hirsutum*, both *GhLAC1* overexpression and RNAi lines exhibited enhanced resistance to *Verticillium dahliae* and cotton bollworm. Interestingly, however, overexpressed *GhLAC1* also increased resistance to cotton aphids, while down-regulated *GhLAC1* showed the opposite. This revealed the critical role of *GhLAC1* in the phenylpropanoid pathway and in the rebalancing of jasmonic acid and salicylic acid [53].

Di et al. (2013) speculated that laccase in *S. miltiorrhiza* might catalyze RA to SalB [34]. Based on the genome sequence of *S. miltiorrhiza*, Li et al. (2019a) identified 29 laccase candidates (SmLAC1-SmLAC29) that contained three signature Cu-oxidase domains [33]. The downregulation of *SmLAC7* and *SmLAC20* in the hairy roots of *S. miltiorrhiza* led to decreased levels of SalB and RA, while their overexpression led to increased levels [33]. In this study, we obtained *SmLAC3* overexpressing transgenic *S. miltiorrhiza* lines and found that the SalB and RA contents were increased in these transgenic lines (Figure 7B). Although these results supported previous speculation that laccase might catalyze RA to SalB, direct in vitro evidence regarding the catalytic activity of the enzyme was lacking. We did successfully obtain purified recombinant SmLAC3 via the prokaryotic expression vector pGEX-4T-1 (data not shown) but failed to obtain the active protein. Efforts should be taken to ascertain the SmLAC3 substrate in the future.

In *Arabidopsis*, lignin synthesis was found to be blocked in the lac11 lac4 lac17 triple mutant, which suggested that these three laccases may be involved in lignin polymerization [30]. In the ornamental plant *Cleome hassleriana*, *ChLAC8* was verified to have a promoting effect on the polymerization of C-lignin [54]. However, our results indicated that the overexpression of *SmLAC3* decreased lignin synthesis in the roots of *S. miltiorrhiza* (Figure 8). Since lignin and phenolic acids share a common upstream phenylpropanoid pathway (Figure 1), we speculated that the decrease of lignin in the *SmLAC3*-OE lines might be caused by the reduction of metabolic flux into the lignin biosynthesis branch due to the significant increase of phenolic acid biosynthesis.

### 3.3. SmLAC3 Is the Target of Sm-miR408

Degradome sequencing and 5’ RLM-RACE are widely used for verifying miRNA cleavage sites. MiR408 often targets genes encoding copper-binding proteins, such as laccase (LAC) and plantacyanin. In *Arabidopsis*, *LAC3*, *LAC12*, and *LAC13* were predicted and validated by 5’ RLM-RACE as target genes of miR408 [15]. Plantacyanin (TC116986) was identified to be the target of Mtr-miR408 in *Medicago truncatula* following 5’ RLM-RACE analysis [22]. Moreover, the study found that dlo-miR408-3p targeted *DlLAC12* in *Dimocarpus longan* Lour [55]. 

Li et al. (2019b) verified that three members (*SmLAC3*, *SmLAC18*, and *SmLAC28*) of the 65 *LAC* gene family in *S. miltiorrhiza* were the target of miR408 via degradome sequencing, and two members (*SmLAC3* and *SmLAC18*) were simultaneously verified by a 5’ RLM-RACE experiment [56]. In the present study, qRT-PCR revealed that the transcript levels of *Sm*-miR408 and *SmLAC3* showed an obvious opposite trend, and 5’ RLM-RACE confirmed that *SmLAC3* is an authentic target gene of miR408 (Figure 4). It must be mentioned that our *SmLAC3* was named according to the 29 laccase members reported by Li et al. (2019a) and that the sequence of *SmLAC3* was consistent with the *SmLAC28* of the 65 members.

## 4. Materials and Methods

### 4.1. Experimental Materials

Seeds of *S. miltiorrhiza* were surface-sterilized, as described previously [57]. Sterile *S. miltiorrhiza* seedlings and transgenic *S. miltiorrhiza* were cultured on a MS basal medium under standard light and temperature growth conditions (22 °C light intensity 100 µmol m^−2^.s^−1^, 16/8 h light/dark cycle). All chemicals were purchased from Sigma Chemical Co. (St. Louis, USA). Solvents were of high-performance liquid chromatography (HPLC) grade. The standards (RA, SalB) used for HPLC were from the National Institute for the Control of Pharmaceutical and Biological Products (Beijing, China). The relevant primers are listed in Table 2.

### 4.2. Construction of Transgenic Vectors and Plant Transformation

The pMDC123SB-AtMIR390a-B/c [58] vector was used to construct the amiRNA-mediated miR408-suppressed expression vector. A pair of amiRNA oligonucleotide primers, amiR-*MIR408*-F/R, were designed using P-SAMS software (http://p-sams.carringtonlab.org/, accessed in 25 January 2018). These synthesized amiRNA oligonucleotide primers were annealed, and the formed cassette was then inserted into the pMDC123SB-AtMIR390a-B/c vector via *BsaI* digestion.

For the *SmLAC3* overexpression vector, the full-length open reading frame (ORF) was amplified with primers 207-*SmLAC3*-F/207-*SmLAC3*-R, which contained *att*B1/*att*B2 sites, and was subsequently recombined into the pDONR207 vector by the standard BP reaction. The entry vector pDONR207-*SmLAC3* was transferred into the vector pEarleyGate 202 (Earley et al., 2006) by an LR reaction to generate the pEarleyGate 202-*SmLAC3* vector, according to the protocol of the Gateway manufacturer (Invitrogen, Carlsbad, USA).

All the recombinant constructs were transformed into *S. miltiorrhiza* using the *Agrobacterium*-mediated transformation method [57].

### 4.3. Molecular Detection of Transgenic Plantlets

To detect miR408-suppressed lines and *SmLAC3*-OE lines at the DNA level, the 2 × 35S CaMV promoter and CaMV35S promoter were amplified, respectively, from genomic DNA, and all the transgenic lines were identified at the DNA level as described previously [59].

The total RNA was isolated from the leaves of two-month-old transgenic plantlets using a Plant RNA Kit (OMEGA, United States). For the expression profiles of *SmLAC3*, the total RNA was isolated from different plant components (e.g., taproot, lateral root, fibrous root, stem, leaf, corolla, calyx, stamen, and pistil) of two-year-old *S. miltiorrhiza* at the flowering stage. The RNA was converted to first-strand cDNA with the PrimeScript^TM^ RT Master Mix (TaKaRa, Beijing, China) according to the manufacturer’s protocols. The transcript levels of the enzyme genes and precursor miR408 were analyzed using qRT-PCR, which was performed with the SYBR Premix Ex Taq^TM^ (TaKaRa, Beijing, China) on a Roche LightCycler^®^96 System. *SmUbiquitin* served as the internal reference. The relative expression of the gene was calculated using the comparative C_T_ method [60].

For the transcript level of mature miR408, the reverse transcription of the total RNA was performed using a Mir-X^TM^ miRNA First-Strand Synthesis Kit (TaKaRa, Beijing, China).

### 4.4. RLM-RACE

The *S. miltiorrhiza* plantlets under a Cu-deficient condition were used for RNA extraction. The 5’ RLM-RACE adapter was obtained through RNA processing following the instruction book. Subsequently, the cDNA acquired by reverse transcription was used as the template for nested PCR according to the protocol of the FirstChoice^®^ RLM-RACE kit (no. AM1700; Invitrogen). The cloned products were inserted into pMD19-T (TaKaRa, Dalian, China) and then sequenced.

### 4.5. Determination of Total Phenolic and Total Flavonoid

The roots of the two-month-old transgenic lines and the control lines were air-dried at 20 ± 2 °C. The contents of the total phenolic and total flavonoid in the roots were measured according to a previous method [61]. 

### 4.6. Detection of RA and SalB by HPLC

The levels of RA and SalB were determined from the roots of two-month-old plants. The phenolic acids were extracted and determined using an Agilent ZORBAX SB-C18 column (250 × 4.6 mm, 5 μm) on an UltiMate 3000 HPLC System (Thermo Fisher Scientific) equipped with a PDA detector as described previously [61].

### 4.7. Lignin Detection by Histochemical Staining

To observe the lignin content changes in different lines, histochemical staining was used to locate and analyze the lignin content. Roots were collected from 60-day-old transgenic lines and control plantlets, and small root segments (5 mm long) were cut from the same parts and immersed in a FAA fixation solution for more than 24 hours followed by paraffin embedding. Subsequently, the sections were stained by means of the saffron staining method using the following steps: First, the sections were soaked in a 1% saffron water solution for about two hours and were rinsed with distilled water and then 75% ethanol several times, and the slices were sealed with 50% glycerin. Finally, the processed sections were observed under a visible light microscope.

The air-dried roots of the three-month-old transgenic and control lines were used to determine the lignin content. Contents of Klason lignin (acid-insoluble) and acid-soluble lignin were measured according to a previous protocol [61].

### 4.8. Determination of Anthocyanin Concentration

The leaves of the two-month-old transgenic and control lines were collected and air-dried at 20 ± 2 °C. The anthocyanin concentration was determined according to previous protocols [62].

## 5. Conclusions

Knocking down *Sm*-miR408 or overexpressing *SmLAC3* in *S. miltiorrhiza* significantly increased the content of SalB and RA. *SmLAC3* was verified as the authentic target of *Sm*-miR408. Although the substrate catalyzed by *SmLAC3* is unknown, our findings indicated that the miR408-*SmLAC3* module participates in SalB synthesis in *S. miltiorrhiza*.

## Figures and Tables

**Table 1:** Lignin contents in the roots of the *SmLAC3*-OE lines and the control.

Line	Lignin (mg/g DW)	
	Klason Lignin	Acid-Soluble Lignin
Control	72.13 ± 2.31	0.53 ± 0.01
OE-2	63.99 ± 3.26	0.47 ± 0.02
OE-10	59.62 ± 3.05	0.45 ± 0.02
OE-15	50.96 ± 1.71 *	0.38 ± 0.01 *
OE-17	52.48 ± 2.14 *	0.41 ± 0.03 *

Data are mean values and standard errors (means ± SDs). Significant differences were determined by one-way ANOVA test relative to the control value (* *p* < 0.05).

**Table 2:** List of primers used for PCR.

Primer	Oligo Sequence 5′ to 3′
amiR-*MIR408*-F	TGTATCTGTCTCGTCCCCGTCTCCAATGATGATCACATTCGTTATCTATTTTTTTGGAGACGGGTACGAGACAGA
amiR-*MIR408*-R	AATGTCTGTCTCGTACCCGTCTCCAAAAAAATAGATAACGAATGTGATCATTGGAGACGGGGACGAGACAGA
RT-*SmUbiquitin*-F	ACCCTCACGGGGAAGACCATC
RT-*SmUbiquitin*-R	ACCACGGAGACGGAGGACAAG
RT-*MIR408*-F	ACAGAAAATGGAGGCGAAGAAG
RT-*MIR408*-R	GTCCCTAATCAGTGAGAGACACAGTAA
RT-*SmLAC3*-F	TGTTGCTCCTTTGGATGCCT
RT-*SmLAC3*-R	CCGTGATCGTGTTGTGGGTT
RT-*SmCYP72A327*-F	GCTACCAACAAGGCAGAAGGAT
RT-*SmCYP72A327*-R	CCTCTCTTCCCATCGCTTCC
RT-*SmBRI-like3*-F	GAAGCAGTTCGCATTCGTGA
RT-*SmBRI-like3*-R	GACCCGAGAGTTGATTGTAGGA
*SmLAC3*-101	GGATACATCCCATTCACCGTGATC
*SmLAC3*-152	ATAACCTTCACCACCAGAGTGTCG
*SmLAC3*-217	CCCATGCACTTCTCATTTGCCT
*SmLAC3*-F	ATGGAGAAGAAGATGAGCTCTTTG
*SmLAC3*-R	TCAACAAAGGGGAAGATCTGG
RT-*TAT1*-F	CGAGCAGGGATGGGAGGTTG
RT-*TAT1*-R	GCCTCTTGGCTGTCTCAGCA
RT-*PAL1*-F	GATAGCGGAGTGCAGGTCGTAC
RT-*PAL1*-F	CGAACTAGCAGATTGGCAGAGG
RT-*PAL2*-F	GGCGGCGATTGAGAGCAGGA
RT-*PAL2*-R	ATCAGCAGATAGGAAGAGGAGCACC
RT-*HPPR1*-F	TGACTCCAGAAACAACCCACATT
RT-*HPPR1*-R	CCCAGACGACCCTCCACAAG
RT-*C4H1*-F	CCAGGAGTCCAAATAACAGAGCCG
RT-*C4H1*-R	GCCACCAAGCGTTCACCAAGAT
RT-*4CL1*-F	TCACCCATGCCGGATTCGAG
RT-*4CL1*-R	AGATCGCGCCGATGAAGGAG
RT-*4CL2*-F	TCGCCAAATACGACCTTTCC
RT-*4CL2*-R	TGCTTCAGTCATCCCATACCC
RT-*RAS1*-F	CCAAAGTCAATTATGCCAAGGG
RT-*RAS1*-R	GTCGGATAGGTGGTGCTCGT
RT-*RAS2*-F	ACTCGGTTCAAATGCGGTAG
RT-*RAS2*-R	GGGCTGGTATTCGTCGTG
RT-*CYP98A14*-F	CCAATCCTACGGCCCGATCC
RT-*CYP98A14*-R	GCCGTCTCTGCTGAGCTTGA
RT-*CCR*-F	CTGATGTTGCTTCGCCTTCT
RT-*CCR*-R	CATACGTGCCTTCCCCTTG
RT-*COMT*-F	GCCACTAAGAATGTTGTCC
RT-*COMT*-R	TCTGTCCTTTCCTTACCA
